# Ill health and distraction at work: Costs and drivers for productivity loss

**DOI:** 10.1371/journal.pone.0230562

**Published:** 2020-03-31

**Authors:** Piotr Bialowolski, Eileen McNeely, Tyler J. VanderWeele, Dorota Weziak-Bialowolska

**Affiliations:** 1 Department of Environmental Health, Sustainability and Health Initiative (SHINE), Harvard T. H. Chan School of Public Health, Boston, MA, United States of America; 2 Department of Epidemiology, Harvard T. H. Chan School of Public Health, Boston, MA, United States of America; Xiamen University, CHINA

## Abstract

Employer-sponsored health insurance is the most widely spread form of medical coverage in the United States. Substantial portion of the premiums’ costs is covered by employers, thus contributing to labor costs for organizations. Although worker health and well-being have become increasingly important for businesses, most of them do not see a direct link between their health and well-being investments and work output and quality of work of their employees. This study aimed to estimate the cost of inefficiencies at work with emphasis on their internal causes, i.e., sick-related absenteeism and distraction at work. With data from 3,258 employees (2,775 office and 483 manufacturing) from a major US manufacturer with revenue of $6 billion, monetary loss in productivity due to sick-related absenteeism and distraction among office and factory floor employees was assessed. The Work Productivity and Activity Impairment scale and the Health-related Lost Productivity Time tool (both already validated) were used to estimate the cost of productivity loss. Survey data on health-related absenteeism and distraction time at work, together with company pay records, were used. A secondary analysis, using survey data collected from 615 Polish apparel factory workers at a major global brand complemented with their payroll records (absenteeism and salary), was conducted to validate the main findings. Results of the primary analysis indicated that annual productivity loss to the organization amounted to approximately $300 m. Distraction contributed to 93.6% of the annual productivity loss of the US manufacturer, while only 6.4% resulted from health-related absenteeism, implying that distraction at work cost this organization almost 15 times more than health related absenteeism, reducing the overall return on sales by over 6 pp. The secondary analysis corroborated the dominance of distraction induced productivity costs over the cost of health-related absenteeism. Evidence from the regression analysis conducted on cross-sectional data indicated that regardless of the type of work, work engagement and auditory privacy were evidently highly bound with productivity loss. For manufacturing workers, job security was also negatively correlated with productivity loss, while for office employees, better social relationships and lack of work-family conflict were positively associated with productivity. Despite being based on two case studies, our results are informative of the magnitude of distraction and health related productivity costs. They also show that workers with deficiencies in their well-being at work present a substantial opportunity for growth to companies in terms of reduced efficiency.

## Introduction

Employees who do not stay on task are costly. They instigate production delays and compel companies to maintain capacity above the optimal level. Alongside major inefficiencies arising from the external environment (e.g., payment delays, bureaucracy, corruption), internally generated costs are an important factor too. Significant sources for costs are health-related absenteeism [1,2], presenteeism (working while sick) [3], and distracted employees [4,5].

Illness affects both work output and quality of work. Regarding the former, absenteeism due to illness reduces worker’s contribution to a firm’s output and profits [2] and can be expressed in monetary value that takes into account a worker’s wage and cost of replacement for the absent worker [6]. Regarding the latter, sick employees experiencing such health conditions as depression, migraine, low back pain, and diabetes, among others, who nevertheless attend work, may perform less efficiently, make more mistakes and contribute substantially to health related labor costs [1,7].

Distracted employees report stress, frustration and lack of motivation [8]. Overall, chatty co-workers and office noise are reported as distractors by 70% and 80% employees respectively, while smartphone use is the number two distraction for younger employees with 69% of the worker population affected [4,8].

Health-related absenteeism and distraction (due to both illness and other distractors) unambiguously lead to a productivity loss. While there have been attempts to assess the cost of illness, usually using the wage rate to estimate the benefit of reduced absenteeism [6] and focusing on the illness specific absenteeism (see for example [1,9–11]), to the best of our knowledge there have been no attempts to estimate monetary value of the productivity loss related to distraction.

Worker health and well-being becomes increasingly important for businesses [12] but as reported by Kyle et al. [13], less than one out of two businesses has a perception of positive return on their health and well-being investments. Consequently, similarly to trends in positive psychology [14,15], more evidence on positive impacts of business engagement in actions to promote health and well-being is needed. In the following sections we present evidence that caring for workers’ well-being beyond pure corporate social responsibility can lead to decreased labor costs since workers with deficiencies in their well-being (while at work and in life in general) present a considerable challenge to companies in terms of reduced efficiency.

Specifically, our study aims to estimate the costs of productivity loss due to health-related absenteeism and distraction at work as well as their correlates. Stewart and colleagues [1,9] found that a majority of costs related to the lost productive time incurred by employers due to either non-communicable diseases or pain experienced by their employees is explained by reduced performance while at work and not by their work absence. Following these findings, we hypothesised that the costs of productivity loss due to health-related absenteeism will be lower than distraction-induced costs.

Unique survey data collected in June 2018 from 3,258 office and manufacturing employees at a major US manufacturer along with the official remuneration records, afforded assessment of the monetary equivalent for distraction and health-related absenteeism. It also enabled identification of work- and human flourishing-related factors associated with the productivity loss. A secondary analysis, using a different dataset (data collected in February 2019 from 615 Polish apparel factory employees at a major global brand), was conducted to provide a robustness check and validate the primary findings.

## Materials and methods

### Data

Well-being Survey (WBS) that comprehensively assesses human flourishing at work and the state of working conditions was used. The WBS was tested on a sample of over 13,000 garment workers in China, Cambodia, Mexico, Sri Lanka, Poland and the United States and of over 5,500 office and manufacturing employees of two Fortune 500 manufacturing companies in the U.S. [16,17].

Data from the two samples of working adults were used. As the primary analysis, we used 3,258 survey responses from the employees (2,775 office and 483 manufacturing) in a major US manufacturer with revenue of $6 billion, yielding a 38% response rate from the original cohort who received an invitation to the survey. Current US based employees of the manufacturer aged at least 18 years of age were eligible to participate in the survey. Employees from outside the US were excluded from the study.

As a secondary analysis, aiming to corroborate the findings, data collected in February 2019 from 615 Polish apparel factory workers at a major global brand (yielding a 100% response rate from the workforce who was invited to the survey and 78% coverage rate from the total workforce) were analyzed. All current employees aged at least 18 years of age were eligible to participate in the survey.

Both studies were preceded by a communication campaign one week prior to data collection (an e-mail with the support sent from the company HR department followed by an e-mail from the research team). Focus of the campaign was to invite employees to participate in the survey. The survey was administered online via Qualtrics to give participants a secure, anonymous space to report on sensitive health topics, including sexual harassment. This survey administration mode was similar to that used by recent high-profile studies [18] and ensured access to adaptable online questionnaire formatted for smart phones and tablets.

Additionally, in the case of primary analysis, wage mid points for annual salaries of exempted employees and hourly wage for hourly paid workers were merged with the survey data (for hourly paid workers incomes were annualized based on average working hours per year provided by the Bureau of Labor and Statistics). In the case of secondary analysis, company records regarding both remunerations and health-related absent days (in the primary analysis these data were self-reported) were merged with the survey data. Individual data were available only to the research team and were not shared with company management, neither were any specific information or analyses allowing employee identification.

All participants provided their written informed consent prior to enrollment in the study. The data collection protocols (with respect to both survey and organization data) were reviewed and approved by Harvard T. H. Chan School of Public Health Institutional Review Board (protocol IRB18-0802 for the primary analysis and protocol IRB14-3500 for the secondary analysis).

Since collected primary sample was slightly overrepresented in favor of females and white employees, all results were weighted to render them representative for the whole company. Weighting variables included gender, age group, race and job tenure. Regarding the secondary sample, the collected data, due to a high coverage and the complete response rate, were closely matching the population structure and thus no weighting was necessary.

### Measures

The Work Productivity and Activity Impairment scale (WPAI) [10,11,19,20] and the Health-related Lost Productivity Time tool [1,9] were adapted to estimate the cost of productivity loss. Both instruments had been validated and used to quantify work productivity and activity impairment by combining the self-reported amount of time missed from work with the self-reported amount of reduced productivity/performance while at work.

Consequently, variables used for our study comprised: (1) self-assessed distraction time at work (% of a workday), (2) either self-reported health related absenteeism measured in days (primary analysis) or health related absenteeism from the company records (secondary analysis) and (3) details of remuneration for relevant employees—provided by the company.

Total productivity loss was calculated in two ways: (i) percentage of working time, (ii) equivalent yearly gross income (in USD) according to the formulae:

$$\begin{array}{l} \mathrm{Total}\;\mathrm{productivity}\;\mathrm{loss}\;(\%)=\mathrm{number}\;\mathrm{of}\;\mathrm{absent}\;\mathrm{days}\;\mathrm{due}\;\mathrm{to}\;\mathrm{health}\;\mathrm{problems}\;(\mathrm{out}\;\mathrm{of}\;\mathrm{last}\;30\;\mathrm{working}\\ \;days)/30+number\;of\;non‐absent\;days(out\;of\;the\;last\;30\;working\;days)/30*distraction\\ \;factor \end{array}$$ (1)

$$\begin{array}{l} Total\;cost\;of\;productivity\;loss\;(USD)=\;number\;of\;absent\;days\;due\;to\;health\;problems\;during\;the\;last\;30\;working\\ \;days/30*gross\;annual\;compensation*multiplier+number\;of\;non‐absent\;days\;(out\;of\;the\\ \;last\;30\;working\;days)/30*distraction\;factor*gross\;annual\;compensation \end{array}$$ (2)

where: the distraction factor is the self-assessed percentage of time at work when the respondent feels distracted (0%, 1–5%, 6–10%, 11–25%, 26–50%, 51–100%; midpoints were used for the analysis); number of absent days due to health problems (per 30) is the productivity loss due to health-related absenteeism; number of non-absent days*distraction factor is productivity loss due to distraction at work.

Additionally, following the reasoning of Nicholson et al. [2] that the cost associated with missed work varies between different positions reflecting the ease with which a manager can find a perfect replacement for the absent worker and can consequently be higher than the wage in these jobs, we applied wage multipliers for non-residential construction workers (1.09) and general office employees (1.30) in the primary analysis, as proposed by Nicholson et al. [2]. The *multiplier* is defined as the cost to the firm of an absence and it is a proportion (often greater than one) of the absent worker’s daily wage. Regarding the secondary analysis, no multiplier was used. This resulted from the fact that the company usually operates below full capacity, receives long-term orders that can be shuffled depending on workers’ availability and by work design trains employees to work on multiple positions to circumvent occupational hazards related to prolonged body postures, repetitive movements, as well as to physical risk factors associated with work in an apparel factory [21]. This implied a negligible absent employee replacement cost.

Total productivity costs, in terms of yearly gross income, were calculated as the average of products of productivity loss measured at the employee level (due to health-related absenteeism and due to distraction at work) taking into account gross annual compensation of employees. In the primary analysis, for exempted employees, annual salaries were directly available from company records, while for hourly paid workers, incomes were annualized based on average working hours per year provided by the Bureau of Labor and Statistics. In the secondary analysis, annual salaries for each employee originated directly from company records.

### Statistical analysis

Mean values for productivity loss and cost of productivity loss were first computed. In the primary analysis the computations were for employees by type of work (manufacturing vs. office), job tenure (up to one year, 1–5 years, more than 5 years), gender and age group (below 30, 30–39, 40–49, 50–59, 60 or more). In the secondary analysis, the stratification was by gender and age group only because apparel factory workers accounted for more than 95% of the surveyed population; additionally, the workforce comprised only employees with a long job tenure (those with the job tenure of more than 5 years accounted for 97.5% and those with the job tenure of 1–5 years–for 2.1%). Two-tailed two sample mean difference test without the assumption of equal variances or analysis of variance was used to assess the significance of observed differences.

Secondly, linear regression was run to establish factors linked with productivity loss. The following factors were tested:

- work engagement–as measured by the Utrecht Work Engagement Scale–and job satisfaction as work outcomes strongly linked to performance [22–24],
- physical working conditions, i.e., visual and acoustical privacy as well as comfortable working position to account for physical demands of work which requires prolonged work postures, repetitive movements, and physical hazards that may significantly influence health, productivity, and performance [25–28],
- psychosocial working conditions, i.e., job control (I have a lot of choice in deciding how I do my work, yes vs. no), mental job demand (I have too much to do to do a good job, yes vs. no), fair treatment (Employees feel they are treated fairly, yes vs. no) and social support from co-workers (I can depend on my coworkers for help, yes vs. no) at workplace since there are theoretical foundations and empirical evidence that they correlate with work outcomes [12,29–32],
- work-family conflict (Demands of my job interfere with my home life, yes vs. no) because it has been theoretically and empirically shown to have a detrimental impact on different work outcomes such as, for example, intent to leave or ability to work [33–40],
- job security (I worry about losing my job, yes vs. no), as it was shown to be correlated with worker well-being and thus potentially impactful for productivity [41],
- human flourishing dimensions: life satisfaction and happiness, purpose in life, close social relationships, character strengths and financial security (measured on a 0–10 scale) after VanderWeele [42], since there is empirical evidence that they correlate with work outcomes [16,17,43,44]. The first two dimensions, i.e., life satisfaction and happiness as well as purpose in life, comprise elements of eudemonic and hedonic well-being [45–47] and positive affect [48]. The third one–close social relationships–corresponds to social well-being [49] and the last one–to financial well-being [50]. The character strengths dimension, suggested by VanderWeele [42,51], is less frequently used.

Gender, age, education, marital status, having children at home, and job tenure (only in primary analysis) served as control variables.

To assesses the degree of multicollinearity among variables [control variables and factors listed in (i)–(vi)], the variance inflation factors (VIFs) [52] for each regression were computed. VIFs ranged from 1.03 to 3.51 and were substantially below 10 –the value recommended by Hair [52] as a threshold for multi-collinearity–and thus multicollinearity was not considered further.

In the primary analysis, models were applied to subsamples of employees working in manufacturing and in office, while in the secondary analysis one regression model was estimated on the sample of textile manufacturing employees only. The outcome was productivity loss, expressed as a percentage of potential loss in productivity against employees with zero distraction and no absenteeism. The regression estimates were then translated into monetary values. In the primary analysis, they were translated into USD using the average annual compensation in the analyzed cross section; in the secondary–into Polish Zloty (PLN) using the actual annual compensation (from the company records) for each employee. Stata 15 was used for analysis.

## Results

Characteristics of the study samples are presented in Table 1 In the primary analysis, participants presented with a mean age of 41.7 years (33% were between ages 18–34, 24% were between ages 35–44, 24% between 45 and 54 and 20% were over the age of 54) and a mean job tenure of 10.6 years. 45.5% of our sample was female, 64.2% was married, 40.4% had at least one child under 18 years old and 4.7% took care of an elderly at home. Over 90.0% had completed at least some college; 88.5% were white.

**Table 1:** Characteristics of participants.

Characteristic	Primary sample (%)	Secondary sample (%)
Gender		
Females	45.5	70.2
Males	55.5	29.8
Age–mean (SD)	41.7 (12.7)	43.3 (7.8)
18–34	33.2	13.8
35–44	23.5	41.3
45–55	23.7	36.1
55 and above	19.7	8.7
Marital status		
Married	64.2	74.5
Single, never married	22.1	8.5
Divorced	8.4	8.2
Widowed	0.7	3.0
Separated	0.8	0.8
Living with a non-married partner	3.8	5.0
Having children under the age of 18 years old	40.4	61.7
Taking care of an elderly	4.7	39.5
Race		
White	88.5	100
Asian	3.3	0
Black	2.8	0
Hispanic	4.1	0
Other	1.3	0
Education		
High school diploma or equivalent	10.0	79.2
Some collage but no degree	18.8	0
Associate degree	12.8	0
Bachelor degree	44.2	7.8
Graduate degree	14.2	12.9
Job tenure–mean (SD)	10.6 (11.2)	15.3 (7.6)
Up to 1 year	15.6	0.3
More than 1 and up to 5 years	32.7	2.1
More than 5 years and up to 10 years	11.0	21.6
More than 10 years	40.7	75.9

Numbers represent percentages unless indicated differently. Unweighted results. N = 3258 (primary sample) N = 615 (secondary sample).

In the secondary sample, the average age of participants was 43.3 (14% were between ages 18–34, 41% were between ages 35–44, 36% between 45 and 54 and 9% were over the age of 54) and a mean job tenure of 15.3 years. 70.2% of the sample was female, 74.5% were married, 61.7% had at least one child under 18 years old and 39.5% took care of an elderly at home. Over 20.7% had completed at least some college; all participants were white.

Our results on the primary sample indicate that average productivity loss due to health-related absenteeism amounted to $679 for office employees and $459 for manufacturing employees, while distraction-induced productivity losses were $10,086 and $6,703, respectively. These numbers indicate that for office employees the distraction induced cost of productivity loss was almost fifteen times higher than the costs related to health-related absenteeism. For manufacturing employees, this ratio was almost at the same level and the costs related to distraction exceeded the costs related to health-related absenteeism more than fourteen times. The analysis of the secondary dataset corroborated a significantly more important role of cost resulting from distraction at work compared to the costs driven by the cost of health-related absenteeism. With the average productivity loss due to health-related absenteeism amounting to 808 PLN (corresponding to 1.84% of the working time) and distraction-induced productivity loss of 3,727 PLN (corresponding to 8.49% of the working time), this ratio–though only for apparel factory employees–was over five in favour of distraction related costs. Note in these analyses, the multiplier (in this study always above 1) was only applied to health-related absenteeism, and thus without its use, the ratio for distraction induced productivity costs over absenteeism, would have been ever greater.

From these figures, yearly productivity loss for a turnover of $6 billion reported by a U.S. manufacturing company was estimated at almost $41 million for manufacturing and $282 million for office employees, yielding a yearly organizational productivity loss of almost $323 million. Of this, average productivity loss due to health-related absenteeism amounted to $16 million, compared with $307 million due to distraction-induced productivity loss. The organization reported an annual turnover of $6 billion, which implies that costs related to productivity loss reduced their return on sales by over 6 pp.

For the Polish apparel factory employees, yearly productivity loss was estimated at over 3.5 million PLN (almost $1 million dollars) with an average productivity loss due to health-related absenteeism amounting to 637 thousand PLN and over 2.9 million PLN due to distraction-induced productivity loss.

When analyzed by type of work, job tenure, age group and gender (primary analysis, Table 2), differences in the average USD costs of productivity loss due to health-related absenteeism were not significant. However, differences in the costs of distraction expressed in USD and as a percent of working time turned out to be statistically significant. Higher disruption costs were generated by males, employees with longer job tenure and employees aged 35–54. The only exception was type of work (office vs. manufacturing), for which no significant difference in the percentage of time distracted was detected. However, since office workers earn much higher salaries, the cost associated with distraction of office workers was significantly higher.

**Table 2:** Average productivity loss and average cost of productivity loss by work type, job tenure, gender and age group–primary analysis of a US manufacturing company.

		Productivity loss–health related absenteeism		Productivity loss due to distraction		Total productivity loss	
		Percent of working time	Equivalent yearly gross income in USD	Percent of working time	Equivalent yearly gross income in USD	Percent of working time	Equivalent yearly gross income in USD
Type of work	Office	0.77*	678.6	12.46	10085.6*	13.23	10764.1*
	Manufacturing	1.11*	459.3	12.59	6703.2*	13.7	7162.5*
Job tenure	Up to 1 year	0.95*	503.4	12.52*	6436.1*	13.47*	6939.5*
	More than 1 up to 5 years	1.04*	816.8	13.73*	9436.9*	14.77*	10253.7*
	More than 5 years	0.68*	570.8	11.71*	10367.8*	12.39*	10938.6*
Age groups	18–35	1.04	684.2	13.75*	7975.0*	14.79*	8659.3*
	35–44	0.77	605.7	12.87*	11005.0*	13.64*	11610.7*
	45–54	0.75	682.8	13.37*	11593.3*	14.12*	12276.1*
	55 or more	0.68	556.6	8.99*	7726.6*	9.67*	8283.2*
Gender	Male	0.67*	598.0	12.96*	10581.5*	13.64*	11179.5*
	Female	1.13*	697.6	11.60*	7531.9*	12.73*	8247.8*

* Starred numbers indicate significant differences at a significance level of 0.05 according to the analyzed characteristic.

In the case of the secondary analysis (Table 3), when analyzed by age group and gender, differences in the average costs of productivity loss due to distraction were not significant. Yet, differences in the costs of health-related absenteeism expressed in PLN and as percent of working time were statistically significant for gender (but not for age groups). Contrary to the primary analysis, higher disruption costs were found to be generated by females.

**Table 3:** Average productivity loss and average cost of productivity loss by gender and age group–secondary analysis of an apparel factory belonging to a global brand.

		Productivity loss–health related absenteeism		Productivity loss due to distraction		Total productivity loss	
		Percent of working time	Equivalent yearly gross income in PLN	Percent of working time	Equivalent yearly gross income in PLN	Percent of working time	Equivalent yearly gross income in PLN
Age groups	18–35	2.5	959.5	11.3	4411.2	13.7	5370.7
	35–44	2.1	855.9	9.0	3758.0	11.1	4614.0
	45–54	1.9	817.3	8.6	3668.0	10.5	4485.3
	55 or more	0.5	348.7	4.3	2825.5	4.9	3174.2
Gender	Male	0.94*	474.9*	6.96	3525.1	7.90	4000.0
	Female	2.35*	959.8*	9.35	3818.5	11.70	4778.3

* Starred numbers indicate significant differences at a significance level of 0.05 according to the analyzed characteristic; Stratification by work type and job tenure was not conducted because 98.5% of the workforce are factory workers and office employees account for only 1.5% of the workforce; 97.6% of employees are employed for more than 5 years.

In the primary analysis, for both office and manufacturing employees work engagement and auditory privacy were factors significantly limiting productivity loss (Table 4). Regarding the work engagement, its increase by 1 point (on a 0–6 scale) was associated with a decrease in the cost of productivity loss of 1,487.8 USD per year for manufacturing and of 2,646.6 USD per year for office employees. As for auditory privacy, if it was ensured, lower loss in productivity was observed–by 2,802.5 USD per year for manufacturing and by 2,345.3 USD per year for office employees.

**Table 4:** Contribution to productivity loss and cost of productivity loss at the organizational level–primary analysis of a US manufacturing company.

Variable	Manufacturing employees			Office employees		
	Standardized regression estimates (95% CI)	Unstandardized estimates (95% CI)	Equivalent yearly gross income in USD	Standardized regression estimates (95% CI)	Unstandardized estimates (95% CI)	Equivalent yearly gross income in USD
Work outcomes						
Work engagement (0–6)	-0.286** (-0.477, -0.095)	-0.038** (-0.063, -0.013)	-1487.8 (per 1 point increase on 0–6 scale)	-0.267*** (-0.317, -0.217)	-0.036*** (-0.042, -0.029)	-2646.6 (per 1 point increase on 0–6 scale)
Job satisfaction (0–10)	-0.027 (-0.167, 0.111)	-0.002 (-0.012, 0.008)	-	-0.046 (-0.104, 0.060)	-0.003 (-0.008, 0.001)	-
Physical working conditions						
Visual privacy (ref. = no)	-0.0012 (-0.097, 0.093)	0.001 (-0.060, 0.057)	-	-0.015 (-0.080, 0.051)	-0.009 (-0.050, 0.031)	-
Auditory privacy (ref. = no)	-0.127** (-0.205, -0.049)	-0.072** (-0.115, -0.028)	-2802.5 (yes vs. no)	-0.056** (-0.093, -0.018)	-0.031** (-0.053, -0.010)	-2345.3 (yes vs. no)
Comfortable position (ref. = no)	-0.004 (-0.105, 0.096)	-0.003 (-0.073, 0.066)	-	-0.037 (-0.122, 0.050)	-0.026 (-0.085, 0.032)	-
Psychosocial working conditions						
Job control (ref. = no)	0.030 (-0.029, 0.091)	0.011 (-0.011, 0.033)	-	-0.034 (-0.134, 0.066)	-0.012 (-0.048, 0.024)	-
Mental job demands (ref. = no)	0.113 (-0.041, 0.268)	0.037 (-0.013, 0.088)	-	0.029 (-0.007, 0.066)	0.009 (-0.002, 0.021)	-
Fair treatment (ref. = no)	0.035 (-0.031, 0.100)	0.013 (-0.012, 0.039)	-	-0.015 (-0.090, 0.060)	-0.006 (-0.035, 0.023)	-
Co-worker support (ref. = no)	0.030 (-0.034, 0.095)	0.015 (-0.017, 0.048)	-	0.023 (-0.039, 0.086)	0.012 (-0.020, 0.043)	-
Work-family conflict						
Work-family conflict (ref. = no)	0.053 (-0.066, 0.172)	0.017 (-0.022, 0.056)	-	0.109*** (0.066, 0.152)	0.036*** (0.021, 0.050)	2647.6 (yes vs. no)
Job security						
Worry about becoming unemployed (ref. = no)	0.147* (0.037, 0.258)	0.054* (0.013, 0.095)	2109.2 (yes vs no)	0.033 (-0.067, 0.134)	0.012 (-0.024, 0.049)	-
Human flourishing						
Life satisfaction and happiness (0–10)	-0.077 (-0.371, 0.216)	-0.008 (-0.036, 0.021)	-	-0.020 (-0.073, 0.032)	-0.002 (-0.007, 0.003)	-
Purpose in life (0–10)	-0.093 (-0.268, 0.082)	-0.008 (-0.024, 0.007)	-	0.013 (-0.072, 0.098)	0.001 (-0.006, 0.009)	-
Character strength (0–10)	0.008 (-0.159, 0.175)	0.001 (-0.016, 0.017)	-	-0.025 (-0.079, 0.029)	-0.002 (-0.008, 0.003)	-
Close social relationships (0–10)	0.008 (-0.336, 0.351)	0.001 (-0.026, 0.027)	-	-0.096* (-0.169, -0.024)	-0.007* (-0.012, -0.002)	-547.9 (per 1 point increase on 0–10 scale)
Financial security (0–10)	-0.050 (-0.266, 0.166)	-0.003 (-0.014, 0.009)	-	0.015 (-0.067, 0.096)	0.001 (-0.003, 0.005)	-

***p<0.001

**p<0.01

*p<0.05; regressions were run controlling for gender, age, education, marital status, having children at home and job tenure.

Work-family conflict was found to be linked with an increase in the costs related to productivity loss among office employees by 2,647.6 USD per year. Out of the five flourishing dimensions, as defined by VanderWeele et al. [51], only close social relationships correlated with reduced productivity loss (by 547.9 USD per 1 point increase on 0–10 scale) and only among office employees. Among manufacturing workers job insecurity emerged as a correlate of productivity declines. When present, it was associated with a 2,109.2 USD per year increase in the cost of productivity loss. All other psychosocial working conditions (job demands, job control, fair treatment and co-worker support) were found to be insignificant among both factory and office workers.

In the secondary analysis conducted for apparel factory workers (Table 5), work engagement and job insecurity emerged as the only correlates of productivity declines, mostly confirming the results of the primary analysis. Change by one point in the work engagement measured on the scale from 0 to 6 was associated with the reduction in the productivity loss due to distraction and health related absenteeism by 703.0 PLN (approximately 180 USD). Job insecurity, when experienced, was associated with a 1,335.0 PLN (about 350 USD) increase per year in the cost of productivity loss. These factors were also found to be significantly associated with the productivity loss for manufacturing employees in the primary analysis. Contrary to the primary analysis, auditory privacy was not identified as a factor associated with the productivity loss but it was only due to the fact that data on auditory privacy were not collected in the secondary analysis.

**Table 5:** Contribution to productivity loss and cost of productivity loss at the organizational level–secondary analysis of an apparel factory belonging to a global brand.

Variable	Standardized regression estimates (95% CI)	Unstandardized estimates (95% CI)	Equivalent yearly gross income in PLN
Work outcomes			
Work engagement (0–6)	-0.160* (-0.316, -0.004)	-0.017* (0-.034, -0.000)	-703.0 (per 1 point increase on 0–6 scale)
Job satisfaction (0–10)	-0.073 (-0.229, 0.084)	-0.005 (-0.015, 0.006)	-
Physical working conditions			
Comfortable position (ref. = no)	0.067 (-0.045, 0.178)	0.012 (-0.008, 0.032)	-
Psychosocial working conditions			
Job control (ref. = no)	-0.047 (-0.152, 0.057)	-0.008 (-0.027, 0.010)	-
Mental job demands (ref. = no)	0.087 (-0.013, 0.188)	0.019 (-0.003, 0.040)	
Fair treatment (ref. = no)	0.043 (-0.067, 0.153)	0.015 (-0.023, 0.053)	-
Co-worker support (ref. = no)	-0.086 (-0.195, 0.023)	-0.022 (-0.050, 0.006)	-
Work-family conflict			
Work-family conflict (ref. = no)	0.025 (-0.084, 0.135)	0.006 (-0.020, 0.031)	-
Job security			
Worry about becoming unemployed (ref. = no)	0.103* (0.002, 0.204)	0.032* (0.001, 0.064)	1335.0 (yes vs no)
Human flourishing			
Life satisfaction and happiness (0–10)	-0.059 (-0.196, 0.078)	-0.006 (-0.018, 0.007)	-
Purpose in life (0–10)	-0.030 (-0.174, 0.115)	-0.003 (-0.016, 0.010)	-
Character strength (0–10)	-0.057 (-0.172, 0.059)	-0.006 (-0.019, 0.006)	-
Close social relationships (0–10)	0.040 (-0.077, 0.158)	0.003 (-0.006, 0.012)	-
Financial security (0–10)	-0.058 (-0.044, 0.160)	0.003 (-0.002, 0.009)	-

***p<0.001

**p<0.01

*p<0.05; regressions were run controlling for gender, age, education, marital status and having children at home.

## Discussion

Although currently employer-sponsored health insurance is the largest source of coverage in the United States, with the average annual premiums amounting to $6,896 for a single coverage and $19,616 for family coverage [53,54], we found that distraction at work cost the manufacturing firm almost 15 times more than health related absenteeism, reducing the overall return on sales by over 6 pp. Specifically, while average productivity loss due to health-related absenteeism amounted to $679 for office employees and $459 for manufacturing employees, the distraction-induced productivity losses were $10,086 and $6,703, respectively. The prevalence of distraction induced costs over the costs related to health-related absenteeism was confirmed in the secondary analysis. Specifically, we found that for apparel factory workers of a global brand distraction at work cost over 5 times more than health related absenteeism. The more pronounced effect in our primary analysis might be attributed to a different pay scheme of workers in the U.S. manufacturer. Workers from the company studied in the secondary analysis were mostly paid according to the piece rate scheme, which has been proven to lead to higher productivity and larger effort [55,56]. Our findings allowed us to confirm our research hypothesis about the greater importance of distraction induced costs over the costs of health-related absenteeism. Similarly to Stewart and colleagues [1,9], we found that the lost productive time and productivity costs caused by work absence are a few times lower than limited performance while at work. Obviously, our approach and theirs differ as ours focused on distraction and theirs–on either specific non-communicable diseases or pain but all three studies unanimously pointed to the fact that most of the limited productivity costs are explained by reduced performance while at work, thus are mostly invisible to employers.

The discrepancy between distraction and health related costs points to a huge potential for improvement in combatting distraction. Although companies cannot insure against distracted employees, they can work towards improvement of work-related factors–especially more engaging work, and auditory privacy–as they are likely to reduce productivity loss. Additionally, for office employees work-family conflict emerges as a significant impediment damaging to productivity, while job insecurity increases distraction among factory workers. Organizations should also consider tailored policies to reduce the considerable costs of productivity loss with respect to distractions among specific group of employees. Our results from the primary analysis suggest that the focus could be on office employees, males, long-term employees, and employees aged 35–54 as distraction of those employees leads to the highest negative effects in the U.S. company. However, the secondary analysis indicated that female employees are more prone to distraction, suggesting that groups prone to distraction should be considered company specific.

This study, despite providing insightful results into the situation of two firms, requires a follow-up analysis of drivers of productivity loss in different settings. First, it would be beneficial to corroborate the principal role of work engagement and auditory privacy for both office and factory workers, as well as the specific substantial role of job insecurity for factory workers and work-family conflict and social relationships for those working in the office. Second, our use of cross-sectional data restrained us from drawing causal inferences. Third, our use of self-reported data on distraction while at work might influenced the findings. Since these self-reports might be subject to social desirability bias [57,58] and most likely be under-reported by respondents, our results concerning the cost of distraction might be underestimated. This, in turn, implies that the predominance of cost of distraction over the cost of health-related absenteeism might be even higher than reported by us. However, our use of the company records on remunerations and also on absenteeism (in the secondary analysis) is not only our contribution to the literature (since so far the costs of lost productive work time due to an illness have been estimated with the self-reported data [1,10,19]) but also provides additional element of objectivity of results. Finally, despite controlling for a rich set of individual and job-related characteristics, the results of regression analysis may still be subject to unmeasured confounding by, for example, personality, core self-evaluations and preferences [59].

To the best of our knowledge, this study is the first to examine the cost of distraction with the use of individual data with both survey and organization records. Although it is clear that the capacity utilization cannot be assumed to reach 100% and some level of distraction is inevitable in company functioning, future studies could help to establish reasonable ranges for an attainable level of focus at work. Additional studies should also examine the consequences of presenteeism on focus at work, as the increased distraction could be consequential of momentary or chronic ill health–the factor we were not able to control in our study.
